# Exploring neural mechanisms of Mandarin tone sandhi perception via fNIRS: the role of gesture in multimodal integration

**DOI:** 10.3389/fnins.2026.1680211

**Published:** 2026-04-22

**Authors:** Yezhi Cui, Fengjie Zhai, Guang Han, Zixuan Ren, Yihan Wang, Zhuanping Qin, Pengke Cui

**Affiliations:** 1 School of Foreign Languages, Tianjin University of Technology and Education, Tianjin, China; 2 School of Life Sciences, Tiangong University, Tianjin, China; 3 School of Automation and Electrical Engineering, Tianjin University of Technology and Education, Tianjin, China

**Keywords:** fNIRS, gesture, Mandarin tone sandhi, multimodal integration, prefrontal cortex

## Abstract

**Introduction:**

The use of pitch gestures in teaching Mandarin tone (i.e., gesture-augmented instruction) has been reported to facilitate lexical tone acquisition. However, the neural mechanisms underlying how gestures aid the learning of complex phonological rules, such as tone sandhi, remain unclear. This study investigated whether gesture-based learning enhances tone sandhi perception primarily through multimodal integration or by promoting cognitive control.

**Methods:**

Forty-four Vietnamese-speaking learners were randomly assigned to either a gesture training group or a no-gesture control group. Post-training, participants performed a disyllabic tone discrimination task while functional near-infrared spectroscopy (fNIRS) data and behavioral responses were recorded.

**Results:**

Results revealed significantly greater neural responses in the left dorsolateral prefrontal cortex (L-DLPFC) in the gesture group compared to the control group. Moreover, enhanced L-DLPFC activation was positively correlated with improved behavioral discrimination accuracy. The gesture group also exhibited strengthened functional connectivity between the L-DLPFC and bilateral prefrontal cortices, accompanied by accelerated hemodynamic responses.

**Discussion:**

Collectively, these findings suggest that gesture-assisted learning facilitates tone sandhi mastery primarily by augmenting prefrontal executive control and fostering multisensory integration. This pattern aligns with a weak embodied cognition framework, in which gestures serve as distributed scaffolds that support, rather than replace, abstract tonal representations.

## 1 Introduction

Mandarin tones are characterized by their distinctive tonal features. Different from to intonational languages, Mandarin employs word-level pitch contrasts to differentiate semantic meanings among words that have identical segmental compositions (Xu, 1994). Research has demonstrated that speakers of both tonal and non-tonal languages can be trained to successfully differentiate between Mandarin tones (Hao, 2012; Li and DeKeyser, 2017; Lu et al., 2015). However, the gains from such training often fail to generalize to naturalistic speech environments, primarily due to the prevalent phenomenon of tone sandhi in spontaneous speech (Shi and Liu, 2021; Xu, 2017; Zhang, 2022). Tone sandhi refers to the phonological phenomenon where the tonal contour of syllables undergoes modification during the process of morphological or syntactic connection (Lin and Wang, 1997). In Mandarin Chinese, the third-tone (T3) sandhi rule serves as a quintessential illustration of this phenomenon, and it is frequently regarded as one of the most complex tonal processes for language learners to master (Shi, 2007; Zhang, 2014, 2016). The perception of T3 sandhi involves several cognitive operations, such as “extraction of underlying tonal categories,” “recognition of surface tone modifications,” and “application of phonological rules and conflict resolution.” This intricate online processing mechanism imposes a substantially greater cognitive demand compared to the perception of isolated syllabic tones (Chen et al., 2025). Specifically, perceiving tonal complexity primarily manifests in two critical dimensions: firstly, the contextual dependency of tone sandhi rules necessitates that learners perform real-time monitoring of tonal sequence dependencies rather than processing individual syllables in isolation, thereby disrupting the stable tonal mappings established during single-character tone acquisition (Li et al., 2016). Secondly, the perceptual process must suppress the representational conflict between underlying tones (T3 + T3) and surface tone sandhi forms (T2 + T3), to prevent interference from literal tonal patterns in semantic extraction, thus imposing significant demands on learners phonological working memory and cognitive control faculties (Guo et al., 2022). This challenge is particularly unique for Vietnamese-speaking learners. Vietnamese is a tonal language comprising six tones: level (33), creaky (21), interrogative (312), falling (325), sharp (24), and heavy (32) (Pan, 2008). Each of these tones has tonal values distinct from the four tones in Chinese. Moreover, native Vietnamese lacks tonal sandhi rules (Pham, 2001), which makes the acquisition of Chinese tone sandhi highly complex. Learners must not only suppress interference from their native phonological system but also construct an entirely new phonological transformation framework in their minds. Therefore, developing an effective pedagogical approach to facilitate the internalization of Chinese tone sandhi rules is of significant practical importance. It also offers a novel perspective for understanding the cognitive mechanisms underlying tone acquisition.

Embodied cognitive theory posits that cognition arises from the dynamic interaction among an individual’s sensorimotor capacities, the physical body, and the environment (Borghi and Pecher, 2011). The strong embodiment perspective asserts that sensorimotor systems form the basis of conceptual representations, while the weak embodiment view contends that sensorimotor processes contribute to, but do not fully constitute, conceptual processing (Khatin-Zadeh et al., 2021; Tirado et al., 2018). The present study adopts the weak embodiment stance, which we argue is more suitable for abstract phonological phenomena such as tone sandhi. Within this framework, gestures are regarded as distributed embodied scaffolds that offer sensorimotor support without supplanting abstract tonal representations (Khatin-Zadeh et al., 2024a). A prominent manifestation of this embodied process in learning is the integration of multiple sensory and motor modalities to form coherent cognitive representations (Dick et al., 2009; Macedonia, 2013). Gestures function as a potent tool for multimodal integration, dynamically linking the auditory signal of speech with visuospatial and kinesthetic feedback (Godfroid et al., 2017). In the realm of second-language (L2) lexical tone acquisition, pitch-gestures do not operate in isolation. They physically instantiate abstract pitch contours, thereby creating a unified audiovisual-kinesthetic representation. It is hypothesized that this representation reinforce tonal category boundaries and reduces cognitive load through enhanced sensorimotor engagement (Borghi and Cimatti, 2010; Zhen et al., 2019). For instance, experimental studies involving Mandarin monosyllabic word pairs with tonal contrasts have demonstrated the pedagogical effectiveness of pitch-modulated gestures in strengthening tonal category boundaries (Morett and Chang, 2015). Recent comparative studies have revealed an intriguing hierarchy in multimodal learning strategies. Producing pitch gestures (PP) outperforms observing pitch features (PO) in L2 lexical tone learning, although its efficacy is influenced by tonal complexity and task requirements (Yu et al., 2024). However, while existing research has extensively examined the learning of monosyllabic Mandarin tones, whether multimodal gesture-assisted learning can facilitate the perception of more complex tone sandhi processes remains unexplored area. Thus, the primary objective of this study is to assess the effectiveness of gestures in supporting Vietnamese learners’ perception of Mandarin tone sandhi phenomena.

During multimodal gesture-assisted learning, not only do behavioral changes occur, but significant alterations also take place in the brain. Previous neuroimaging studies have demonstrated that the effects of learning involving bodily actions are most commonly manifested as changes in cortical brain activity at parietal and frontal regions (Kontra et al., 2015; Pelicioni et al., 2019) identified as a particularly crucial area for learning, considering its well-established role in mediating higher-order cognitive functions, such as attention allocation, executive control, linguistic processing, and visuospatial integration (Miller and Cohen, 2001). Research utilizing functional magnetic resonance imaging (fMRI) has revealed that gestures activate the prefrontal cortex, whereas non-relevant movements do not induce such activation (Newcombe, 2010). The dorsolateral prefrontal cortex (DLPFC) plays a pivotal role in regulating intentional actions through its top-down cognitive control mechanisms. These mechanisms actively suppress reflexive or habitual responses to facilitate deliberate decision-making (de Manzano and Ullén, 2012). The ventrolateral prefrontal cortex (VLPFC), on the other hand, integrates multimodal signals through specialized neurons and responds to audiovisual stimuli. This process is of critical importance for communication and language processing (Romanski, 2012). Given the complementary roles of DLPFC in inhibitory control and VLPFC in multisensory integration, investigating the functionality of both regions during behavioral tasks may shed light on their joint contributions to cognitive control and multimodal processing.

Recent studies increasingly employ functional Near Infrared Spectroscopy (fNIRS) to explore the neural correlates associated with complex cognitive tasks, encompassing those involving embodiment and multimodal processing (Lamb et al., 2018; Li and Bodet, 2023; Liu et al., 2025; Watanuki and Asaka, 2012). fNIRS is a non-invasive neuroimaging modality that measures cortical hemodynamic activity by detecting changes in the absorption of near-infrared light by oxyhemoglobin (HbO) and deoxyhemoglobin (HbR) (Pinti et al., 2020). Specifically, near-infrared light is projected onto the scalp, and then the absorption of this light is measured. Hemodynamic activity is inferred from the attenuations in light levels (Quaresima et al., 2012). In comparison to other neuroimaging techniques, such as fMRI and electroencephalogram (EEG), fNIRS stands out due to its portability, cost-effectiveness, safety for repeated use, and strong tolerance to motion artifacts. These characteristics collectively allow for ecologically valid monitoring of cortical activity during dynamic, naturalistic tasks (Hoshi, 2007). These advantages render fNIRS uniquely suitable for investigating real-time cortical dynamics in multimodal learning. A common paradigm in tone perception research is a discrimination task, in which participants are required to determine whether pairs of auditory stimuli are identical or different, thereby evaluating their perceptual sensitivity to tonal contrasts (Baills et al., 2019; Chen et al., 2015; Fu and Evans, 2025; Zhang et al., 2025). Consequently, we employed a disyllabic-tone discrimination task in combination with fNIRS to examine cortical activation patterns during gesture-enhanced multimodal integration.

This study investigates whether gesture-enhanced multimodal learning improves Vietnamese learners’ perception of Mandarin tonal sandhi and explores the underlying neural mechanisms. To this end, we compared behavioral responses and hemodynamic changes detected via fNIRS under two experimental conditions: a gesture condition (incorporating auditory, visual, and kinesthetic modalities) and a no gesture condition (relying on auditory and visual modalities only). We focused on three core questions. The first is whether the gesture group outperforms the control group in Mandarin tone sandhi discrimination tasks. The second pertains to how gesture training affects the activation intensity, temporal dynamics, and functional connectivity of the prefrontal cortex, especially the left dorsolateral prefrontal cortex (L-DLPFC). The third question concerns the relationships between these neural activation patterns and behavioral performance. By integrating behavioral data with neuroimaging findings, this research seeks to shed light on the neurocognitive mechanisms by which multimodal integration, facilitated by embodied gestures, reshapes prefrontal networks, thereby facilitating the acquisition of complex phonological rules.

## 2 Materials and methods

### 2.1 Participants

Forty-four native Vietnamese speakers (29 females; mean age = 21.9 ± 1.2 years) participated in this study. All participants were first-year undergraduate international students enrolled at multiple universities in Tianjin, China. Since Standard Mandarin serves as the primary language for both instruction and daily communication in this metropolitan setting, the participants were immersed in a high-intensity Mandarin-speaking environment through both academic and social interactions.

Participants were recruited through an online background questionnaire (Supplementary Material A) designed to establish eligibility based on the following inclusion criteria: (1) Vietnamese as the sole native language with monolingual upbringing prior to international study; (2) limited proficiency in languages other than Vietnamese (excluding English as the academic lingua franca); (3) possession of a valid HSK4 certification; (4) self-reported right-handedness, normal audiovisual function, and absence of neurological/speech-language disorders or formal musical training.

Only individuals meeting all criteria were invited to participate. Considering temporal variations in HSK certification dates, participants’ current Mandarin proficiency was re-evaluated using the latest official HSK4 test materials 1 week prior to the experiment. Certified language instructors administered and graded all assessments. Eligible participants were then randomly assigned to either the gesture group (n = 22, 8 males, 14 females) or the no gesture group (n = 22, 7 males, 15 females) using a computer-generated sequence. Detailed characteristics of the two groups are presented in Table 1. Independent-samples *t*-tests revealed no significant differences between the two groups in terms of age, HSK4 scores, or learning duration (all p > 0.05), indicating that baseline proficiency and experience were comparable and unlikely to confound the results. All participants provided written informed consent prior to the experiment.

**TABLE 1:** Baseline characteristics of participants by experimental group (M ± SD).

Group	Male	Female	Age	HSK4	Learning duration (months)
Gesture	8	14	21.9 ± 1.2	271.9 ± 7.6	26.7 ± 3.5
No gesture	7	15	21.9 ± 1.3	271.0 ± 8.0	26.6 ± 3.6

HSK4, Chinese Proficiency Test Level 4.

### 2.2 fNIRS data acquisition

**FIGURE 1:**
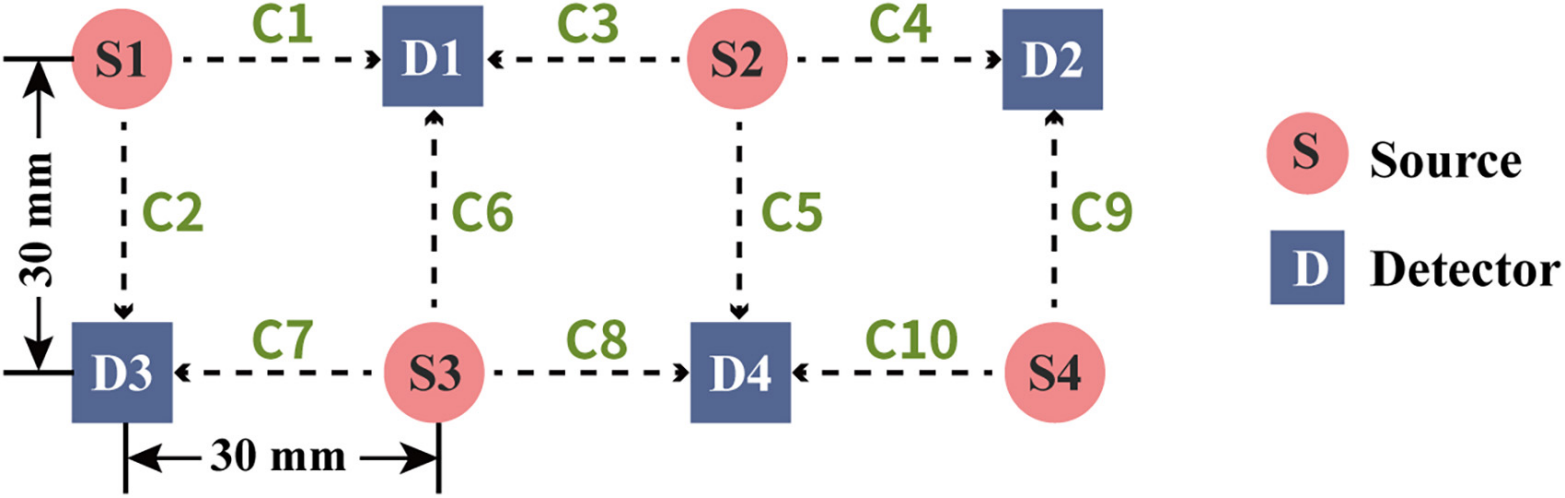
Schematic diagram of probe placement over the prefrontal cortex. The wearable device consists of four sources (red circles, S1–S4) and four detectors (blue squares, D1–D4), forming a total of ten recording channels (green labels, C1–C10). The distance between each source–detector pair is 30 mm.

Functional near-infrared spectroscopy data were acquired using a Brain-Explorer-Wear system (NIRS Ergonomics, China) equipped with two wavelengths (775 nm, 855 nm). This wearable device enabled continuous monitoring of prefrontal cortical activation via relative changes in oxyhemoglobin (HbO) and deoxyhemoglobin (HbR) concentrations (Liu et al., 2021). The sampling rate of the machine was 5 Hz. The acquisition software used was NIRS-App_Wearable, which was developed by Tianjin University. A probe array mounted on the device included 4 sources and 4 detectors with a fixed 30-mm source-detector distance, generating 10 measurement channels. The device was placed on the participant’s forehead, with the center of the band placed approximately on the Fpz location (international 10–20 system) (Dashtestani et al., 2018). The forehead region covered by each channel are shown in Figure 1. All channels were clustered into 6 regions of interest (ROIs), which were the frontopolar cortex (FOA), orbital frontal cortex (OFA), DLPFC, VLPFC on the left (L) and right (R) hemispheres. The standard Montreal Neurologic Institute (MNI) coordinates, as well as the region labels for each channel, are listed in Table 2.

**TABLE 2:** Montreal Neurologic Institute (MNI) coordinates and Brodmann labels of channel cortical projection points.

Channel	MNI			Region	Brodmann label
	x	y	z		
Ch1	−29	52	19.8	L-DLPFC	46
Ch2	−32	62.5	7.2	L-DLPFC	46
Ch3	0	67.5	18.8	FOA	10
Ch4	29	52	19.8	R-DLPFC	46
Ch5	−12	69	8.5	FOA	10
Ch6	12	69	8.5	FOA	10
Ch7	−29	57	−2.7	L-VLPFC	47
Ch8	0	72	−2.7	OFA	11
Ch9	32	62.5	7.2	R-DLPFC	46
Ch10	29	57	−2.7	R-VLPFC	47

MNI, Montreal Neurologic Institute; L-DLPFC, left dorsolateral prefrontal cortex; FOA, frontopolar cortex; R-DLPFC, right dorsolateral prefrontal cortex; L-VLPFC, left ventrolateral prefrontal cortex; OFA, orbital frontal cortex; R-VLPFC, right ventrolateral prefrontal cortex.

### 2.3 Materials

We constructed disyllabic word lists, containing 16 words (with 4 words for each of the four third-tone sandhi patterns; see Table 3). These words were selected based on two operational criteria: (1) Instructional-Level Matching: all words were drawn from the standardized HSK Level 3 and Level 4 vocabulary lists, ensuring lexical alignment with the participants’ formal proficiency. (2) Controlled Frequency: the written frequency of each word was verified using the BCC Corpus^1^. Words with extremely high or low frequency were excluded to balance cognitive accessibility and avoid automatic processing or excessive difficulty. Chinese has four lexical tones, traditionally labeled T1, T2, T3, and T4. The phonetic characteristics of these tones are as follows: T1 (high-level, 55), T2 (high-rising, 35), T3 (low-dipping, 214), and T4 (high-falling, 51) (Chen et al., 2014). Mandarin Tone 3 sandhi in disyllabic compounds manifests in four tonal combinations: T3 + T1, T3 + T2, T3 + T3, and T3 + T4. Mandarin T3 sandhi serves as a prime illustration of tonal alternation. This phonological process exhibits two categorical patterns: (1) When a T3 syllable (with an underlying tone of 214) precedes another T3, the first T3 undergoes a tonal shift from its canonical low-dipping contour (214) to a high-rising pitch pattern (35) that phonetically approximates T2, as in the word 你 好/ni^214^ + hao^214^/→/ni^35^ + hao^214^/; (2) When followed by non-T3 tones, the T3 syllable reduces to a low-level tone (21), known as the half-third sandhi, exemplified by 北京/bei^214^ + jing^55^/→/bei^21^ + jing^55^/. It is worthwhile mentioning that the surface phonetic realization of the half-third tone can be more complex and context-dependent than a categorical 21 (Yuan and Chen, 2014); nevertheless, for the purpose of creating clear and pedagogically effective stimuli in this study, we adopted this standard, categorical representation.

**TABLE 3:** List of disyllabic words used in T3 sandhi experiments.

Combination	Word			
T3 + T1	shoudû	jiandân	xiaoshuô	binggân
T3 + T2	qilái	dazhé	xuanzé	yiqián
T3 + T3	xizao	jianshao	lijiì	dasao
T3 + T4	baohù	manyì	liwù	bisài

T1, T2, T3, and T4 represent the four tones in Mandarin Chinese: T1 is the first tone (high-level), T2 is the second tone (rising), T3 is the third tone (low-dipping or falling-rising), and T4 is the fourth tone (falling).

**FIGURE 2:**
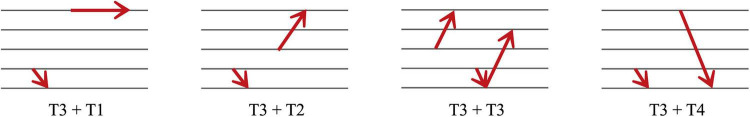
Schematic pitch contours illustrating Mandarin third-tone (T3) sandhi in different tonal contexts. T1, T2, T3, and T4 denote the four lexical tones in Mandarin Chinese: T1 (high-level), T2 (rising), T3 (low-dipping/falling-rising), and T4 (falling). The diagrams show how the pitch contour of T3 changes when it is followed by T1, T2, T3, or T4.

These phonological principles, along with the traditional five-scale tone chart, were employed to generate dynamic pitch contour visualizations in Microsoft PowerPoint 2021. To enhance cross-modal learning, standard Pinyin orthography was positioned beneath the animations as orthographic cues. All auditory stimuli were recorded by a female native Chinese speaker using Cool Edit Pro 2.1 (Adobe Systems Incorporated) with a sampling rate of 44100 Hz. Each word had a duration of 1000 ms and was normalized to 70 dB via Praat software. Figure 2 illustrates the pitch feature diagrams for each type of Mandarin T3 sandhi.

In disyllabic tone discrimination task, each word was paired with two auditory stimuli: a canonical production adhering to sandhi rules and a deviant variant. Deviant stimuli included two violation types: full sandhi omission (T3 + T3 → T3(214) + T3) and half sandhi over application (T3 + T1 → T2 + T1).

### 2.4 Procedure

The entire experiment was carried out utilizing E-Prime 3.0 (Psychology Software Tools, Inc.) within a sound-proof laboratory. Participants were seated in front of a computer screen, with a viewing distance maintained at 40–45 cm. The video stimuli were presented at the center of the screen, which had a resolution of 1920 × 1080 pixels, and were displayed within a masked area. Considering the viewing distance, these visual dimensions corresponded to a visual angle ranging from 79.61 to 86.30 degrees.

To present the auditory stimuli, we employed a pair of flanking loudspeakers positioned adjacent to the display. Over the course of the entire experiment, we meticulously calibrated the intensity levels of the auditory stimuli and kept them consistently stable. This rigorous calibration and maintenance were carried out with the aim of guaranteeing the accuracy and reliability of the experimental results.

**FIGURE 3:**
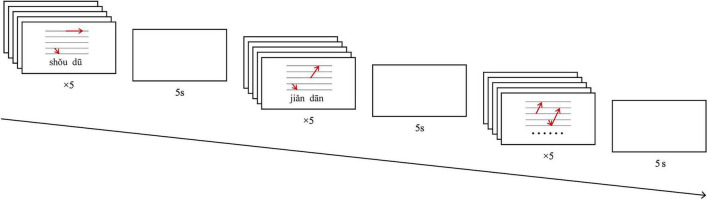
Schematic illustration of a trial sequence in the gesture condition of the tone learning task. In each trial, participants view a video presenting a disyllabic Mandarin word together with an animation depicting the pitch contour of the T3 sandhi pattern. Simultaneously, they hear a native Mandarin speaker’s pronunciation of the word. Participants in the experimental group imitate the pronunciation while tracing the corresponding pitch contour on a touch-sensitive panel with their dominant index finger. Each word is presented five times, separated by a 5-s inter-trial interval. The entire task lasts approximately 15 min per session. Training is conducted three times per week over 3 weeks.

Participants initially undertook the disyllabic word tone learning task (as illustrated in Figure 3). In this task, they were instructed to learn the tones of Mandarin T3 disyllabic words, selected from those listed in Table 3, in preparation for subsequent assessments. During each trial of the task, a single target word sourced from Table 3 was presented via a video. Participants were shown an animation that depicted the pitch contour of the Mandarin T3 sandhi phenomenon for that specific disyllabic word. Simultaneously, they were provided with an audio example of a native Mandarin speaker pronouncing the target word. Participants in the experimental group were required to follow the pronunciation and, at the same time, draw the corresponding tonal contour on a touch-sensitive panel using their dominant index finger. Participants in the control group were presented with the identical video animations and audio examples but were instructed to remain still and refrain from making any gestures while watching and listening. All Mandarin words (from Table 3) were presented in a random order, with each word being presented five times during the word-learning task. After a 5 s inter-trial interval, the trial was repeated with another Mandarin word also chosen from Table 3. The entire word learning task lasted approximately 15 min. Both groups underwent the training three times a week, amounting to a total of 9 sessions over a period of 3 weeks.

**FIGURE 4:**
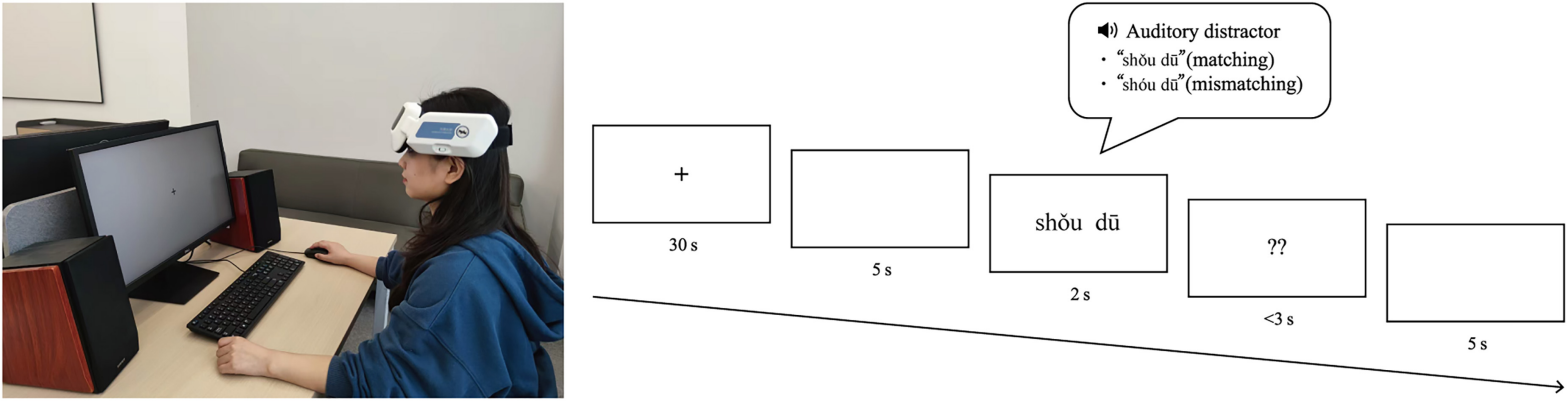
Experimental scenario (left) and trial procedure of the disyllabic tone discrimination task (right). Each trial begins with a fixation cross (30 s), followed by a 5-s blank screen. A target word is then presented auditorily and visually for 2 s. Following stimulus offset, a probe displaying two question marks appears, and participants have 3 s to indicate whether the tone matches the pronunciation by pressing the left or right mouse button. Button assignments are counterbalanced across participants. A 5-s blank screen follows each response. The task consists of 32 trials, with half containing matching stimuli and half mismatched stimuli, randomly interleaved. Each tone-pronunciation combination is equally represented across trial types.

Following the learning task, participants completed the disyllabic tone discrimination task. The experimental scenario and procedure are shown in Figure 4. Each trial started with a 30 s fixation cross at the center of PC monitor, followed by a blank screen (5 s), and then the target word accompanied by its auditory pronunciation (2 s). After the target word disappeared, the probe with two question marks would appear. Participants were instructed to determine whether the previously displayed lexical tone was consistent with the pronunciation by pressing the left or right mouse button as quickly as possible. If no response was recorded within 3 s after the probe appeared, the trial was marked as an incorrect response. Once a response was made, there would be a blank screen lasting for 5 s until the next trial. The assignment of left/right buttons to “match” or “mismatch” responses was counterbalanced across participants. Half of the trials consisted of pronunciations with matching lexical tones, and half of trials consisted of pronunciations with deviant stimuli, with trial types randomly interleaved. A total of 32 trials were presented, with each possible combination of pronunciation and lexical tone being presented an equal number of times within each trial type (matching vs. mismatching).

### 2.5 Behavioral response data analysis

We analyzed the collected behavioral data using R version 4.4.1. Initially, the data underwent preprocessing, during which reaction times (RTs) shorter than 200 ms or longer than 3000 ms were excluded. Such RTs are generally considered to represent ineffective or mechanical responses. Subsequently, mean reaction time (RT) for correct responses and accuracy rate (ACC) were calculated for each participant within their assigned group (experimental or control). All data were normalized to identify and exclude outliers whose standard z-score exceeded 3. After these preprocessing and outlier-exclusion steps, an independent samples *t*-test was conducted to compare the differences in averaged RT and ACC across the two groups.

### 2.6 fNIRS data analysis

Functional near-infrared spectroscopy data were analyzed using the NIRS_SPM package in MATLAB (R2013b) based on the general linear model (GLM) (Ye et al., 2009). Preprocessing steps included hemodynamic response functions (HRF) and Wavelet-MDL method to exclude noise caused by participants’ head movement, heartbeat, and breathing and drift. These two methods have been demonstrated in related studies (Brigadoi et al., 2014; Jang et al., 2009) and are considered to be relatively effective in denoising. The hemodynamic changes for each of the 10 channels were calculated using the modified Beer-Lambert law (Boas et al., 2001).

In our analysis, we exclusively focused on changes in HbO concentration (ΔHbO). Previous studies have demonstrated that HbO signals generally exhibit a larger amplitude and are more sensitive to task-related neural activity compared to HbR signals (Fu et al., 2014; Hoshi, 2003). Consequently, ΔHbO was adopted as the key metric for analyzing the fNIRS results. To investigate the impact of gesture on brain activity, after the fNIRS data were preprocessed, the statistical analysis employed the following methodologies: (1) Brain activation analysis: the task-related β-values under different learning conditions were calculated by GLM as a measure of brain region activation. The β-values from channels within the same anatomical region of interest (ROI) were averaged to obtain a single regional activation strength per participant per condition. A 6 (ROI: L-DLPFC; R-DLPFC; FOA; OFA; L-VLPFC; R-VLPFC) × 2 (Condition: gesture; no gesture) mixed ANOVA tested for main effects and interactions of gesture. (2) Event-related time series analysis: to further investigate the temporal dynamics of gesture modulation, we conducted an event-related hemodynamic time-series analysis. The mean ΔHbO time series within the −2 to 6 s time window was extracted from significantly activated brain regions. For the 0–6 s post-stimulus period, we calculated both the mean and peak activation levels, using the pre-stimulus interval (−2 to 0 s) as the baseline for correction. A linear mixed-effects model was fitted with ΔHbO as the dependent variable. The fixed effects included condition (gesture; no gesture), continuous time variable, and their interactions, and subject as a random factor. (3) Functional connectivity analysis: functional connectivity strength between different brain regions was quantified using the Pearson correlation coefficient. A higher correlation coefficient indicates stronger functional connectivity. All correlation coefficients (*r*-values) were converted to Z-scores using Fisher’s Z-transformation, which served as the final metric for the functional connectivity strength between each pair of brain regions. Between-group differences in these Z-scores were assessed using two independent samples *t*-tests (p = 0.05). (4) Behavior-neuro correlation analysis: Pearson’s correlations were calculated to assess the relationship between behavioral performance in the tone discrimination task and activation intensity (beta values) in significantly activated brain regions. All p-values were corrected using FDR (false discovery rate) and p < 0.05 was considered significant after correction.

## 3 Results

### 3.1 Behavioral response results

An independent samples *t*-test was conducted to compare the differences in averaged RT and ACC between the two learning conditions. The study data exhibited no significant outliers and approximated a normal distribution within each group, with homogeneous variances. The results revealed a significant effect of gesture on ACC, *t*(42) = −6.223, *p* < 0.001. However, the RT data illustrated no significant differences between gesture and no gesture condition, *t*(42) = −1.992, *p* > 0.05. Results are shown in Table 4.

**TABLE 4:** Reaction time (RT) and ACC for no gesture and gesture groups during the disyllabic tone discrimination task.

Measure	No gesture		Gesture	
	Mean	SD	Mean	SD
RT (ms)	598.16	157.08	509.45	137.74
ACC (%)	58.76	16	84.15	11

### 3.2 fNIRS data

#### 3.2.1 Brain activation analysis

**FIGURE 5:**
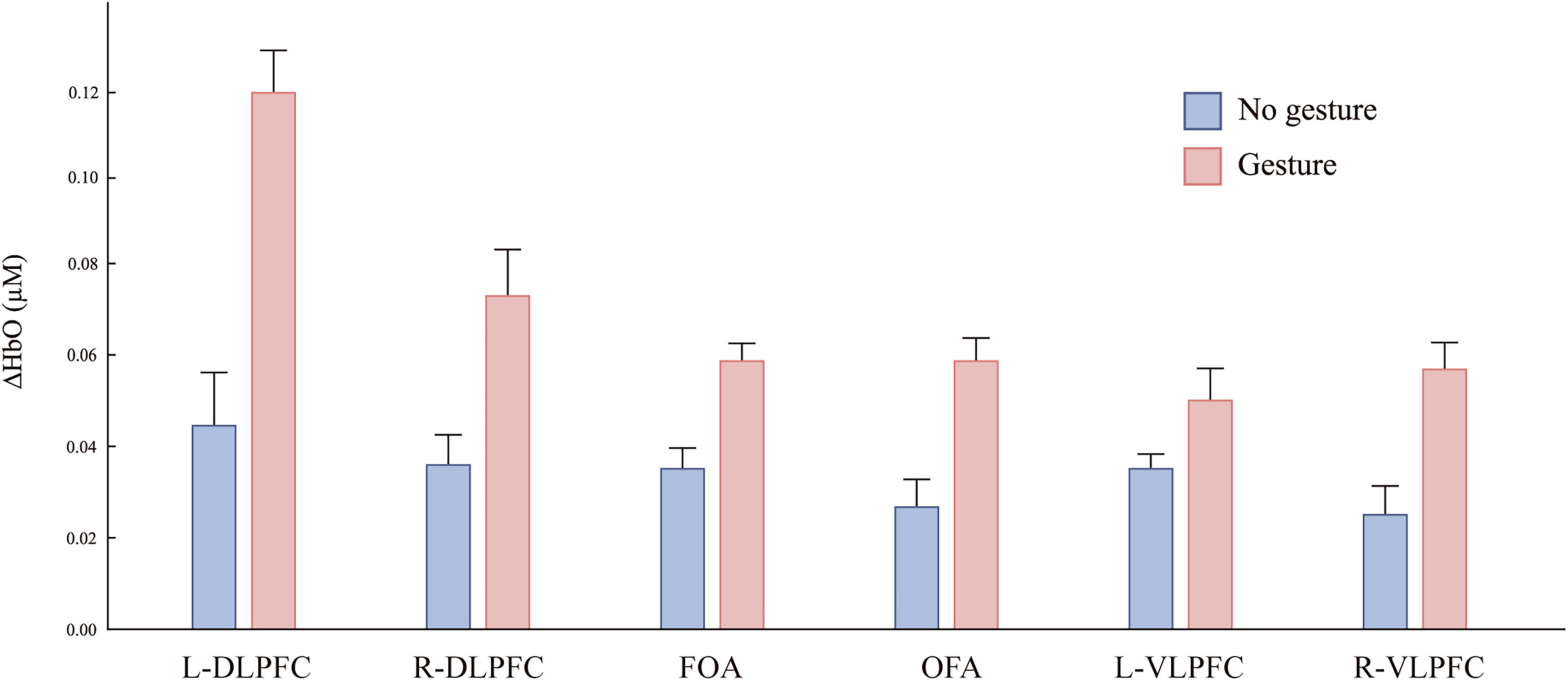
Mean changes in oxygenated hemoglobin concentration (ΔHbO, μM) in prefrontal cortex regions during the disyllabic tone discrimination task under no-gesture and gesture conditions. Bars represent mean ± SEM across six regions of interest (ROIs): left and right dorsolateral prefrontal cortex (L-DLPFC, R-DLPFC), frontopolar area (FOA), orbitofrontal area (OFA), and left and right ventrolateral prefrontal cortex (L-VLPFC, R-VLPFC).

**FIGURE 6:**
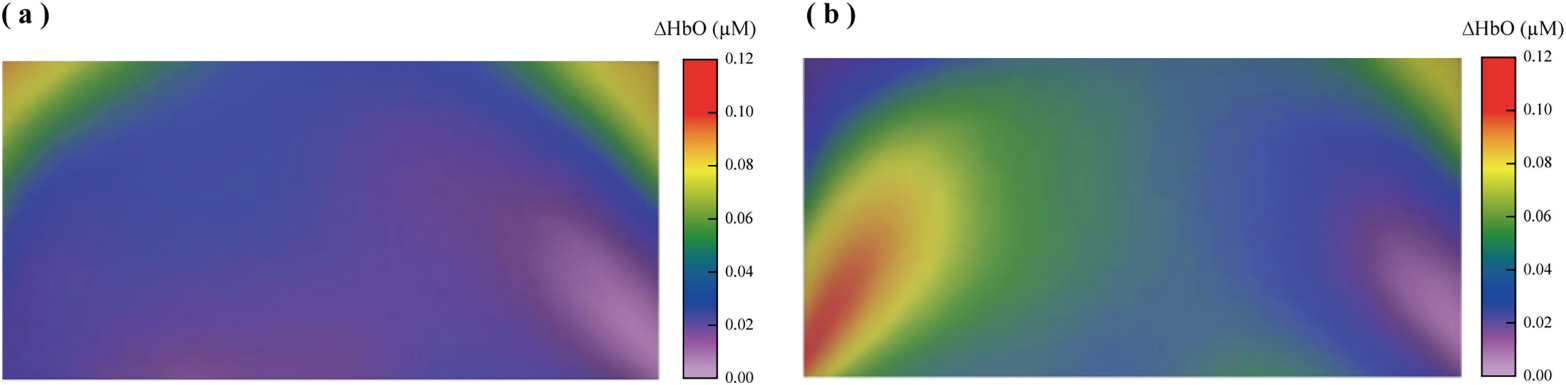
Prefrontal cortical activation patterns during the disyllabic tone discrimination task under no gesture and gesture conditions (FDR-corrected, *p* < 0.05). **(a)** Spatial distribution of significant ΔHbO responses in the no gesture condition. **(b)** Spatial distribution of significant ΔHbO responses in the gesture condition.

The average changes in the hemodynamic responses across prefrontal regions during the gesture and no gesture conditions are depicted in Figures 5, 6. Notably, a substantial difference in the average ΔHbO in the L-DLPFC in Figure 5. Results from the mixed ANOVA on ΔHbO found a significant effect of ROI [*F*(5, 210) = 7.96, *p* < 0.001, η^2^*_*p*_* = 0.159], a significant main effect of condition [*F*(1, 42) = 56.08, *p* < 0.001, η^2^*_*p*_* = 0.57], and that the HbO concentration in the gesture condition was significantly greater than that in the no gesture condition. In addition, there were significant interaction between ROI and condition [*F*(5, 210) = 4.95, *p* < 0.001, η^2^*_*p*_* = 0.105]. To further explore simple effects, we employed a Linear Mixed Effects Model (LMM). The analysis showed that HbO activation in the gesture condition was significantly greater than that in the no- gesture condition in the L-DLPFC [*F*(1, 42) = 33.98, *p* < 0.001, η^2^*_*p*_* = 0.447]. However, there were no significant differences in other prefrontal regions (all *p* > 0.05). Collectively, these findings suggest that gestural interventions specifically enhance activation in the L-DLPFC.

#### 3.2.2 Event-related time series analysis

**FIGURE 7:**
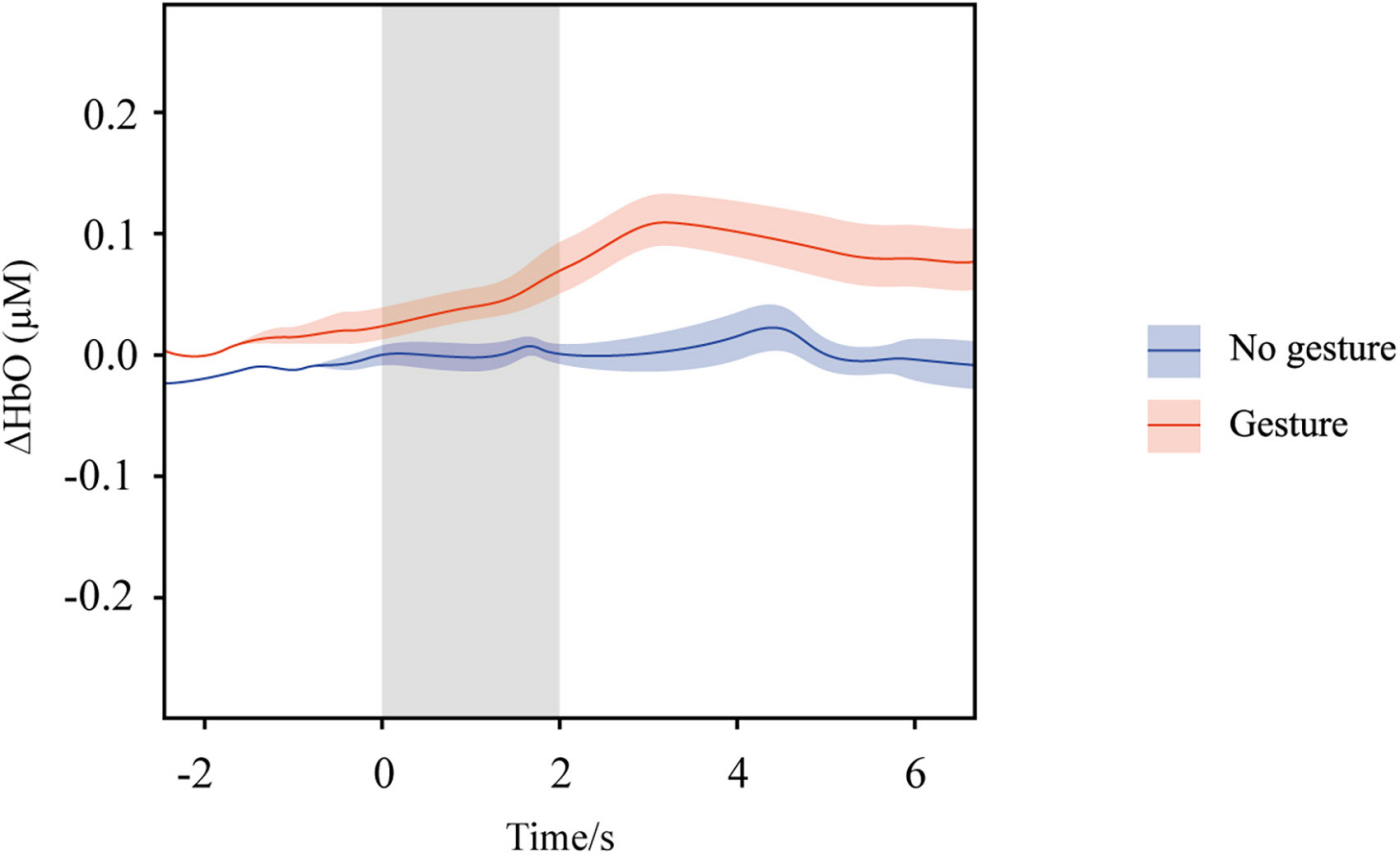
Block-averaged ΔHbO responses in the left dorsolateral prefrontal cortex (L-DLPFC) under the gesture (red) and no gesture (blue) conditions. The gray shaded area indicates the stimulus presentation window (0–2 s). Solid lines represent the mean time courses, and the shaded ribbons indicate ± SEM.

The event-related time series analysis focused on the significantly activated L-DLPFC. Block-averaged ΔHbO responses in the gesture and no gesture conditions in the L-DLPFC are shown in Figure 7. A linear mixed-effects model revealed a significant interaction between time and group (β = 0.015, SE = 0.004, t = 3.75, p < 0.001), indicating that the gesture group exhibited a significantly steeper increase in ΔHbO in the L-DLPFC over time compared to the no gesture group. During the 0–6 s post-stimulus window, the gesture group showed a significantly higher mean ΔHbO activation level (0.052 μM) than the no gesture group (0.030 μM), t(42) = 4.81, p < 0.001, d = 0.95. Concurrently, the time-to-peak activation was significantly earlier in the gesture group (3.0 s) than in the no gesture group (4.3 s), t(42) = 3.45, p = 0.001, d = 0.72. These results suggest that gesturing not only enhanced the activation intensity in the L-DLPFC but also accelerated the time course of its hemodynamic response.

#### 3.2.3 Functional connectivity

**FIGURE 8:**
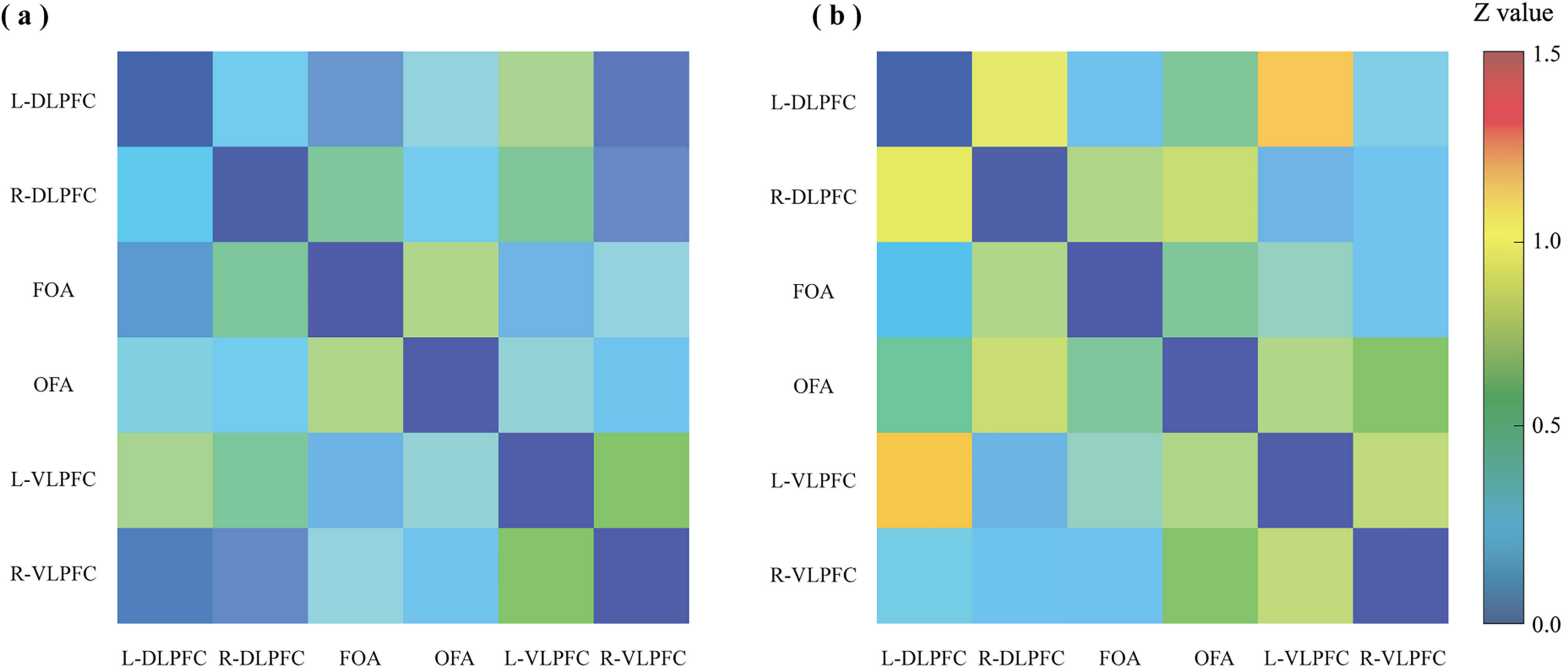
Functional connectivity (FC) matrices for panel **(a)** the no gesture condition and **(b)** the gesture condition during the disyllabic tone discrimination task. The matrices include six regions of interest (ROIs): L-DLPFC, R-DLPFC, FOA, OFA, L-VLPFC, and R-VLPFC. Colors represent Fisher Z-transformed connectivity strength, with warmer colors indicating stronger connectivity.

The functional connectivity matrices for the two groups are shown in Figure 8. Statistical analysis (FDR-corrected) revealed that compared to the no gesture group, the gesture group showed significantly enhanced functional connectivity between L-DLPFC and R-DLPFC, and between L-DLPFC and L-VLPFC (all *p* < 0.05).

#### 3.2.4 Relationship between behavioral data and fNIRS data

**FIGURE 9:**
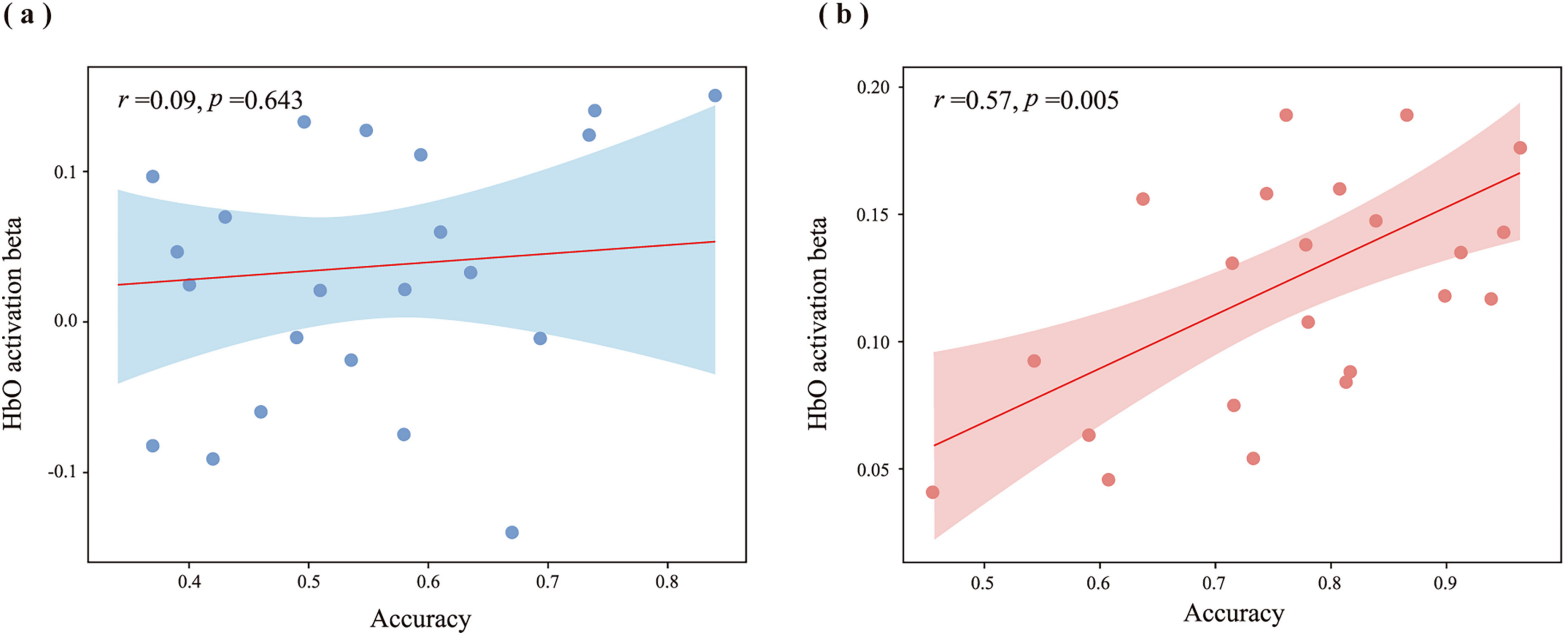
Correlations between accuracy rates and L-DLPFC activation under gesture and no gesture conditions. **(a)** In the no gesture condition, no significant correlation is observed (*r* = 0.09, *p* = 0.643). **(b)** In the gesture condition, a significant positive correlation is observed (*r* = 0.57, *p* = 0.005).

In the only ROI, that is, the L-DLPFC, which showed significantly greater fNIRS responses in the gesture condition compared to no gesture, we further examined whether gestures facilitate tone acquisition through the involvement of the language processing or attention network. The Pearson correlations between HbO activation beta values of in L-DLPFC and ACC, and RT, were calculated in two learning conditions. As shown in Table 5, results using Bonferroni correction showed no significant correlations between response times (RT) and hemodynamic activity in the L-DLPFC under either experimental condition (all *p* > 0.05). In the no gesture condition, ACC (*r* = 0.09, *p* = 0.643) showed no significant association with hemodynamic response magnitudes (beta weights) in the L-DLPFC. Conversely, gesture intervention elicited a robust positive correlation between accuracy rates and L-DLPFC activation (*r* = 0.57, *p* = 0.005) (see Figure 9).

**TABLE 5:** Pearson correlations between ACC, RT and beta values for the gesture and no gesture conditions in the L-DLPFC.

Variable	Statistic	No gesture	Gesture
RT, beta	r	−0.37	0.06
	*p*	0.092	0.782
ACC, beta	r	0.09	0.57
	*p*	0.643	0.005**

ACC, accuracy; RT, reaction time; r, Pearson correlation coefficients; beta values (reflecting hemodynamic activity) in the left dorsolateral prefrontal cortex (L-DLPFC); statistical significance is marked as follows: *p ≤ 0.05, **p ≤ 0.01, ***p ≤ 0.001.

## 4 Discussion

The present study employed fNIRS to investigate the neurocognitive mechanisms underlying gesture-enhanced perception of Mandarin disyllabic tones among Vietnamese-speaking learners. Adopting a weak embodiment framework, in which sensorimotor processes contribute to but do not constitute conceptual representations, we interpret gestures as distributed embodied scaffolds that support tone sandhi perception without replacing abstract tonal codes. Integrating behavioral and fNIRS data, our findings show that gestures selectively enhanced the intensity and temporal dynamics of activation in the L-DLPFC, strengthened functional coupling between the L-DLPFC and bilateral prefrontal regions, and predicted individual differences in perceptual accuracy. These findings indicate that gesture-based learning enhances tone sandhi mastery chiefly through two interconnected mechanisms: strengthening prefrontal executive control and promoting multisensory integration. This explanatory framework aligns with the weak embodied cognition perspective while advancing it by specifying how distributed sensorimotor anchors can reconfigure prefrontal networks to support cross-linguistic phonological acquisition.

### 4.1 The role of gestures in tone sandhi perception: a multisensory integration perspective

From the weak embodiment perspective adopted in this study, gestures do not replace abstract tonal representations but rather provide complementary sensorimotor input that facilitates perceptual processing. The efficacy of gestures in facilitating the perception of monosyllabic tones among L2 learners has been well-established in previous research (Morett et al., 2022; Wong et al., 2007; Zheng et al., 2018). Our behavioral findings also revealed that participants in the gesture condition achieved significantly higher accuracy rates (84.15%) in the disyllabic tone discrimination task compared to those in the no gesture condition (58.76%), despite the fact that the RTs were comparable between the two conditions. This dissociation between improved accuracy and unaltered processing speed suggests that the multimodal enrichment provided by gestures enhances the quality of phonological encoding rather than accelerating responses. A plausible explanation lies in the mechanism by which gestures transform abstract auditory pitch contours into visuomotor cues, thereby creating a multimodal representation of tones (Getz et al., 2017). This process of sensorimotor integration may strengthen phonological encoding by anchoring auditory signals to motor and visual schemas, which in turn stabilizes tonal categories in memory.

While this richer representational format has the potential to enhance perceptual accuracy, it may not necessarily accelerate decision-making, as the integration and retrieval of multimodal information may require additional cognitive resources (Madan and Singhal, 2012). Alternatively, the need to suppress cross-linguistic interference from Vietnamese tonal categories during disyllabic processing could also be a contributing factor. Gestures may mitigate such interference by directing attentional focus to critical tonal distinctions (e.g., rising vs. falling contours) (Goldin-Meadow and Wagner, 2005; Sun, 2016). The cognitive resources required for this inhibitory control could offset potential gains in processing speed, resulting in stable RTs alongside improved accuracy. These behavioral patterns align with the weak embodiment framework.

### 4.2 Prefrontal engagement and network coordination: evidence for an embodied control mechanism

The observation that gesture production during the learning phase is associated with significantly greater activation of the L-DLPFC during the discrimination task offers initial neural evidence indicating that gestures may modulate tone processing in this specific population. Participants in the gesture condition not only demonstrated heightened L-DLPFC activation but also exhibited an earlier peak latency, suggesting a potential shift toward more efficient neural engagement.

This pattern is consistent with the sensorimotor hypothesis, which posits that cognitive processes are deeply rooted in sensory and motor systems. The processing of disyllabic sequences imposes substantial demands on phonological working memory and inhibitory control to manage cross-linguistic interference. The observed L-DLPFC activation likely reflects its role in addressing these dual demands, with gestures serving as a motoric “scaffold” that offloads and organizes this cognitive load. Critically, under the weak embodiment stance adopted in this study, this scaffolding function does not imply that the motor patterns of gestures replace or constitute the abstract phonological representation of tone sandhi. Rather, gestures provide complementary sensorimotor input that facilitates executive control and reduces cognitive load, while the abstract tonal rules likely retain amodal or modality-independent components. A strong positive correlation between L-DLPFC activation and behavioral accuracy (*r* = 0.57, *p* = 0.005) substantiates the interpretation that the engagement of this region is functionally relevant. As part of the frontoparietal control network, the L-DLPFC may optimize attentional allocation for resolving tonal ambiguities (Sacca et al., 2023). Moreover, our results provide insights into how embodied actions may promote large-scale network coordination. The gesture condition was associated with enhanced functional connectivity between the L-DLPFC and the right DLPFC, as well as between the L-DLPFC and the L-VLPFC. The strengthened bilateral DLPFC connectivity implies improved interhemispheric integration for cognitive control, potentially triggered by the spatial properties of the gestures (Passarotti et al., 2002; Shomstein, 2012). Concurrently, the enhanced connectivity between the L-DLPFC and the L-VLPFC indicates the development of an efficient cortical circuitry underlying cognitive regulation.

These findings support the hierarchical prefrontal organization framework (Badre and Wagner, 2007; Kuhl et al., 2008; Scalf et al., 2009), in which the L-DLPFC encodes goal-relevant information (e.g., the target tonal pattern), and the L-VLPFC selects specific features (e.g., pitch contours). From an embodied cognition perspective, gestures may transform abstract pitch goals into spatial-motor patterns, thereby streamlining the interaction between these prefrontal regions and enhancing the efficiency of tonal feature selection within working memory (Wimber et al., 2015). Importantly, the selective engagement of the L-DLPFC within the prefrontal regions sampled in this study aligns with the distributed embodiment framework (Khatin-Zadeh et al., 2023). According to this view, abstract concepts such as tonal rules are not fully grounded in a single sensorimotor modality (e.g., audition) but are instead distributed across multiple brain regions, with prefrontal executive areas coordinating sensorimotor and amodal representations. While our fNIRS montage did not cover temporal lobe regions typically associated with auditory processing, the observed L-DLPFC-specific effect suggests that gesture-based learning may operate primarily through prefrontal executive mechanisms. Gestures provide a distributed sensorimotor anchor for pitch contours, allowing the L-DLPFC to coordinate auditory, visual, and kinesthetic inputs without eliminating the abstract phonological code. This interpretation is consistent with the proposal that gestures serve as a scaffold for executive functions in abstract concept processing (Khatin-Zadeh et al., 2024b).

### 4.3 Neurocognitive mechanisms: toward an embodied, multimodal integration account

Collectively, our findings suggest that gestures were associated with enhanced tone sandhi perception, potentially through multimodal integration, a core tenet of embodied cognition theory. The learning context created by synchronized gesture execution, visual observation, and auditory perception provides complementary sensory inputs that likely reinforce memory encoding more effectively than auditory input alone (Farran and Morett, 2024; Shams and Seitz, 2008). Critically, the gestures were not arbitrary but spatially mapped to tonal contours (Alibali et al., 2011), providing a visuomotor analogue for dynamic pitch changes. This may transform the learning process into an embodied experience, where the physical enactment of pitch variations through hand movements may lead to deeper, motorically-grounded encoding of phonological patterns (Hostetter and Alibali, 2008; Lakoff and Johnson, 2017).

The neurophysiological evidence further substantiates this integrated account. The heightened and temporally-shifted activation in the L-DLPFC suggests its involvement in coordinating kinesthetic, spatial, and auditory information, potentially reflecting its role as a key node within a distributed network for multisensory integration (Plakke and Romanski, 2014). Thus, the effectiveness of gesture-based instruction can be interpreted as stemming from two interconnected mechanisms: it provides broad multisensory enrichment, while the specific, accurate motor encoding of tonal features optimizes the efficacy of this enrichment, aligning closely with both the sensorimotor hypothesis and pedagogical principles of embodied learning.

In conclusion, this study provides preliminary neurocognitive evidence, grounded in a weak embodiment framework, that gesture-enhancement in tone learning may operate through embodied mechanisms that modulate prefrontal activation dynamics and network coordination, with gestures serving as distributed scaffolds that support rather than replace abstract tonal representations. While these interpretations are constrained by the specific sample and study design, they offer a theoretical advancement by linking behavioral outcomes in multimodal language learning to potential underlying neural processes framed within embodied cognition. Future research with larger, more diverse samples and longitudinal designs is needed to confirm these observed relationships and further elucidate the causal pathways involved.

### 4.4 Limitations and future directions

Our study provides illuminating insights into the neurocognitive mechanisms of gesture-enhanced learning in Mandarin tone sandhi acquisition, but recognizes some limitations. First, we randomly assigned participants into two groups. It is better to conduct pre-test tasks like tone discrimination to ensure that the two groups of participants were matched on tone perception capacity before learning. Second, the generalizability of findings is constrained by our focus on young adult Vietnamese learners: a population whose native tonal system partially overlaps with Mandarin. Whether these results extend to naturalistic L2 tone acquisition by speakers of non-tonal or typologically distant tonal languages remains untested. Third, and most critically from a theoretical perspective, our experimental design features a fundamental confound between the effects of embodied learning and those of general multimodal enhancement. The gesture group involved additional motor and somatosensory modalities that were absent in the control group. Consequently, Future research should incorporate an active control condition that involves non-representational motor actions (e.g., rhythmic tapping or arbitrary hand movements) matched for motoric complexity but devoid of pitch contours links to the phonological targets. Such a design is essential to disentangle the unique contribution of embodied mapping from the general effects of motor activation and multimodal integration in tone sandhi learning. Fourth, while the current stimuli targeted the T3 sandhi due to its phonological regularity (Chien et al., 2021), Lin’s work research shows that other disyllabic tone combinations (e.g., T2 + T4) exhibit systematic acoustic variations in prosodic contexts (Lin, 1996). Subsequent research should comparative investigations of neural encoding patterns across diverse disyllabic tone combinations, while systematically examining how gestures modulates phonological processing for L2 learners. A final limitation is that, the examined brain regions in the present study involved the prefrontal areas. While revealing significant results in the L-DLPFC, the study was unable to assess temporal lobe engagement, which is a critical region for auditory and phonological processing (Osnes et al., 2011). Prior studies demonstrate that the superior temporal gyrus (STG) and superior temporal sulcus (STS) encode acoustic-phonetic features of speech (Defenderfer et al., 2017). Future work should integrate neuroimaging techniques (e.g., fNIRS or EEG) with broader cortical coverage to capture temporal lobe dynamics during tone sandhi learning. For instance, investigating how gesture-enhanced training modulates functional connectivity between the temporal lobe (auditory encoding) and premotor areas (articulatory planning) could elucidate the neural mechanisms of cross-modal integration in L2 phonological acquisition.

## 5 Conclusion

This study provides initial fNIRS evidence that gesture-based learning may enhance Mandarin tone sandhi perception in Vietnamese learners. Behaviorally, gestures were associated with improved discrimination accuracy. Neurophysiologically, this advantage corresponded with modulated left dorsolateral prefrontal cortex (L-DLPFC) activation and altered prefrontal network dynamics. These findings align with the weak embodied cognition framework, which posits that sensorimotor processes play a contributing role in conceptual processing but do not form its entirety. Within this view, gestures serve as distributed embodied scaffolds that enhance prefrontal executive control and multisensory integration rather than replacing abstract tonal representations. The observed neural profile suggests that gestures may facilitate learning by fostering integrated multisensory representations of phonological rules, with the L-DLPFC playing a likely coordinating role. Thus, this work provides neurocognitive support for the role of sensorimotor engagement in language learning, offering a preliminary basis for considering gesture-enriched pedagogy in teaching complex phonological structures like tone sandhi. Further research is needed to confirm and generalize these relationships.

## Data Availability

The raw data supporting the conclusions of this article will be made available by the authors, without undue reservation.

## Footnotes

1
http://bcc.blcu.edu.cn/
