# Supplementary material for: Exploring neural mechanisms of Mandarin tone sandhi perception via fNIRS: the role of gesture in multimodal integration
Source: Front Neurosci. 2026 Apr 22;20:1680211. doi: 10.3389/fnins.2026.1680211 (PMC13144161; doi:10.3389/fnins.2026.1680211)
Supplement: Supplementary file 1 [file Table_1.docx]

**Supplementary Material A: Participant Demographic Questionnaire**

Dear Madam/Sir,

Hello! Thank you for taking the time to participate in this survey! The survey does not involve any personal privacy or commercial interests, and is only for scientific research. Your opinion is important to the research, so please read the questions in turn and answer them carefully. Thank you very much for your support and cooperation!

**Part 1: Demographic Information**

1. Participant ID: _________________________
2. Age: _________ (Years)
3. Sex: ○ Male ○ Female ○ Other/Prefer not to say
4. Handedness: ○ Left-handed ○ Right-handed ○ Ambidextrous

**Part 2: Language Background**

1. What is your first/native language? _________________________
2. Apart from first/native language, do you have functional proficiency in any other language(s)?

○ No

○ Yes – If yes, please specify the language(s) and select your approximate level of proficiency for each:

Language Proficiency Level (Please select one per language)

__________ ○ Basic (A1–A2) ○ Intermediate (B1–B2) ○ Advanced (C1–C2)

__________ ○ Basic (A1–A2) ○ Intermediate (B1–B2) ○ Advanced (C1–C2)

1. What is your total cumulative duration of learning Mandarin?

_________ Years _________ Months (Please include all formal and informal learning experiences)

1. Please provide details of your highest HSK certification:

Level Achieved: ○ HSK 1 ○ HSK 2 ○ HSK 3 ○ HSK 4 ○ HSK 5 ○ HSK 6

Score : _________ / 300

**Part 3: Health and Experience Screening**

1. Do you have a history of any of the following? (You may select multiple)

□ Hearing impairment
□ Vision impairment (not corrected by glasses/lenses)
□ Speech or language disorders
□ Learning disabilities (e.g., dyslexia, ADHD)
□ Neurological disorders (e.g., brain injury, epilepsy)
□ None of the above

1. Have you received more than one year of formal music training (e.g., on an instrument or in voice)?

○ Yes
○ No
